# RECIPROCAL RELATIONSHIP BETWEEN COGNITIVE STATUS AND HIERARCHY FUNCTIONAL LOSS AMONG FRAIL OLDER ADULTS IN CHINA

**DOI:** 10.1093/geroni/igz038.2349

**Published:** 2019-11-08

**Authors:** Qian Sun, Nan Jiang, Nan Lu, Vivian W Lou

**Affiliations:** 1 Hebei University of Business and Economics, Shijiazhuang, Hebei, China; 2 Columbia University, New York, United States; 3 Renmin University of China, Beijing, China; 4 The University of Hong Kong, Hong Kong, P.R.C., Hong Kong

## Abstract

The present study aimed to determine the reciprocal relationship between cognitive status and the loss hierarchy of specific functional activities among frail older adults in China. Data were derived from a sample of 469 older adults who participated in both the 2010 and 2013 waves of the Longitudinal Study on Family Caregivers for Frail Older Adults Aged 75 or Above in Shanghai, China. A two-wave cross-lag analysis was used to examine the proposed model. In general, the results showed that cognitive status in 2010 was a significant predictor of dependence in activities of daily living (ADLs) in 2013. Specifically, cognitive status at baseline has significant effects on feeding [β (SD) = -0.198 (0.043), p < .001], continence [β (SD) = -0.172 (0.045), p < .001], bladder [β (SD) = -0.159 (0.045), p < .001], toileting [β (SD) = -0.119 (0.043), p < .001], hygiene [β (SD) = -0.108 (0.044), p < .05], stairclimbing [β (SD) = -0.101 (0.044), p < .05], and dressing in 2013 [β (SD) = -0.100 (0.045), p < .05]). Furthermore, toileting and bathing in 2010 were significant risk factors of cognitive status in 2013 [toileting: β (SD) = -0.146 (0.066), p < .05; bathing: β (SD) = -0.113 (0.047), p < .05]. The findings not only expanded our understandings of the relationship between cognition and the hierarchy of functional loss, but also provided evidences for clinicians and service planners for anticipating the subsequent care and service needs of the elderly and their families.

